# Identification of a Novel Subpopulation of Tumor-Initiating Cells from Gemcitabine-Resistant Pancreatic Ductal Adenocarcinoma Patients

**DOI:** 10.1371/journal.pone.0081283

**Published:** 2013-11-21

**Authors:** Kazuya Shimizu, Sachie Chiba, Yuichi Hori

**Affiliations:** 1 Department of Internal Medicine, Kobe Medical Center, Kobe, Japan; 2 Division of Medical Chemistry, Department of Biophysics, Kobe University Graduate School of Health Science, Kobe, Japan; Thomas Jefferson University, United States of America

## Abstract

Pancreatic ductal adenocarcinoma is highly resistant to systemic chemotherapy. Although there are many reports using pancreatic cancer cells derived from patients who did not receive chemotherapy, characteristics of pancreatic cancer cells from chemotherapy-resistant patients remain unclear. In this study, we set out to establish a cancer cell line in disseminated cancer cells derived from gemcitabine-resistant pancreatic ductal adenocarcinoma patients. By use of *in vitro* co-culture system with stromal cells, we established a novel pancreatic tumor-initiating cell line. The cell line required its direct interaction with stromal cells for its *in vitro* clonogenic growth and passaging. Their direct interaction induced basal lamina-like extracellular matrix formation that maintained colony formation. The cell line expressed CD133 protein, which expression level changed autonomously and by culture conditions. These results demonstrated that there were novel pancreatic tumor-initiating cells that required direct interactions with stromal cells for their *in vitro* cultivation in gemcitabine-resistant pancreatic ductal adenocarcinoma. This cell line would help to develop novel therapies that enhance effects of gemcitabine or novel anti-cancer drugs.

## Introduction

Pancreatic ductal adenocarcinoma (PDAC) is an aggressive malignancy with a high cancer dissemination rate, which results in high mortality [1]. The majority of PDAC patients already have either locally advanced or metastatic cancer, when the patients aware symptoms. Thus, they are treated with mainly gemcitabine- or fluorouracil-based systemic chemotherapy [2,3]. Clinical benefit response was experienced by 23.8% of gemcitabine-treated patients, but the PDAC thereafter got resistant to gemcitabine, resulting in 6 months of median overall survival [2-5]. Understanding how PDAC gets resistant to gemcitabine is important for development of novel therapies that enhance effects of gemcitabine or novel anti-cancer drugs. It is conceivable that characterizations of carcinoma cells derived from gemcitabine-resistant PDAC patients are useful. Such cell lines, however, have not been established, because adjuvant chemotherapy before surgical resection is not common for PDAC and PDAC cell lines reported in previous papers are generally from surgical specimen of PDAC patients who did not receive chemotherapy [6-10]. 

 It was reported that PDAC consisted of heterogeneous carcinoma cells [11,12]. We and other groups reported that there were CD133^+^ carcinoma cells in PDAC [7,13-15]. CD133^+^ carcinoma cells were observed in invasive border zone of PDAC [7,13], and CD133^+^ cells were enriched when PDAC or cultivated cells were treated with gemcitabine [7]. On the other hand, it was reported that there were no CD133^+^ carcinoma cells in PDAC [16]. Because existence of CD133^+^ carcinoma cells in PDAC is a controversial question, characteristics of CD133^+^ carcinoma cells derived from gemcitabine-resistant PDAC patients have not been clarified. 

 In the present study, we for the first time succeeded in establishing a novel CD133^+^ tumor-initiating cell line in disseminated cancer cells derived from gemcitabine-resistant PDAC patients, using *in vitro* co-culture system with stromal cell lines.

## Materials and Methods

This study was performed according to Institutional Review Board-approved guidelines in Kobe Medical Center and Kobe University School of Health Sciences and we obtained approval from Ethics Committees of Kobe Medical Center and Kobe University School of Health Sciences (permission No.152). Written informed consent was obtained from all patients.

### Human Tissue Specimens

Seven patients had diagnosis of advanced PDAC (at Stage IVa or IVb based on the TNM classification for pancreatic cancer) by clinical and radiological reports with evaluation of cytological study of pancreatic ducts in Kobe Medical Center. All the patients were treated with conventional chemotherapy with or without local radiotherapy. We obtained disseminated PDAC cells in carcinoma tissues, peritoneal effusions, or pleural effusions from those patients. A qualified pathologist (M.F.) analyzed the samples.

### Isolation of KMC14 Cells

Peritoneal effusion was obtained from the patient 1 (Table 1). The precipitated cells were washed with phosphate-buffered saline (PBS) and suspended with serum-free Stem medium (DS Pharma Biomedical, Osaka, Japan) containing 0.1 µM 2-mercaptoethanol and 50 U/ml of penicillin and 50 µg/ml of streptomycin (PenStrep) (Invitrogen, Carlsbad, CA). The cells were cultured on the confluent PA6 or TIG3 stromal cells at 37°C in a humidified atmosphere containing 5% CO_2_. Colonies were hand picked under a microscope and re-plated on confluent stromal cells. The colony-forming cells were termed KMC14 cells. For preparation of a single KMC14-cell suspension, KMC14 colonies were hand picked under a microscope, followed by treatment of 0.04 units of Liberase Blenzyme 3 (Roche Diagnostics, Basel, Switzerland) [17]. The cells were re-suspended with serum-free Stem medium and passed through a 40 µm-pore filter (BD Biosciences, Franklin, NJ). The pass-through fraction was used as a single KMC14-cell suspension.

**Table 1:** Summary of patients and their clinical characteristics.

Pat.#.	Sex	Age	Pathol. subtype	Therapy	Metastases	Origin of KMC cells
#1. (KMC14)	F	78	Tub.	Gem: 7 g/m^2^ TS-1: 7.8 g/m^2^	Liver, Peritonea, Ip-LN, Lung, Pleura	Pleural effusion
#2. (KMC16)	F	73	Tub.	Gem: 6 g/m^2^ TS-1: 0.9 g/m^2^	Liver, Peritonea, SV, Ip-LN, Om	Peritoneal effusion Om
#3. (KMC17)	M	57	Tub.	Gem: 14 g/m^2^ TS-1: 2.8 g/m^2^ CRT	Liver, Peritonea. Ip-LN, Lung, Pleura	Peritoneal effusion Pleural effusion, Liver
#4. (KMC18)	F	72	Tub.	Gem: 4 g/m^2^	Liver, Peritonea, Om, Spl. Col., Int. Uterus, Ip-LN, Dia.	Peritoneal effusion, Liver, Om
#5. (KMC26)	M	80	Tub	Gem: 20 g/m^2^	Liver, Peritonea, Ip-LN, Om	Peritoneal effusion
#6. (KMC07)	M	69	Tub.	Gem: 71 g/ m^2^ CRT	Liver, Peritonea, Ip-LN	Peritoneal effusion
#7. (KMC09)	M	67	Tub.	Gem: 14 g/m^2^	Liver, Stomach, Spl., Dia. GB, Ip-LN	Liver

Pat. #., Patient number; Pathol. subtype, Pathological subtype; Tub., Tubular adenocarcinoma; Gem, gemcitabine; TS-1, a 5-fluorouracil derivative; CRT, the 5-fluorouracil (250 mg/m^2^/day for 28 days) chemotherapy with radiation therapy (50.4 Gy/28 fractions); Ip-LN, intraperitoneal lymph nodes; Om, omentum; Spl, spleen; Col, colon; Int, intestine; SV, splenic vein; Dia, diaphragm; GB, gallbladder.

### Stromal and Pancreatic Cancer Cell Lines

Mouse stromal cell line PA6 was supplied from Dr. Nishikawa (RIKEN, Kobe, Japan)[18]. Human embryonic stromal cell line TIG3 was supplied from the Japan Health Sciences Foundation (Tokyo, Japan) [19]. Human pancreatic cancer cell lines, BxPC-1, PANC-1, MIAPaCa-2 and AsPC-1 cells, were obtained from American Type Culture Collection (Rockville, MD).

### Preparation of Cytology Specimens

Cultured cells were fixed in 4% paraformaldehyde (PFA) (Wako, Tokyo, Japan) for 5 minutes. Both cells and extracellular matrix (ECM) were harvested using a cell scraper (IWAKI, Tokyo, Japan). After centrifugation, the precipitant was suspended in liquefied HistoGel (Thermo Fisher Scientific Inc., Waltham, MA) and allowed to solidify. The HistoGel-embedded samples were soaked in 10% phosphate-buffered formalin for overnight and embedded in paraffin. Deparaffinized and rehydrated 3-µm sections were applied for H&E, Alcian-Blue and Azan staining. For frozen sections, the HistoGel-embedded samples were frozen in OCT compounds and thin-sliced by a cryostat. The 10-µm sliced samples were fixed in 95% Et-OH, followed by immunohistochemistry.

### Immunohistochemistry

Immunostaining for human CD133 was performed with a mouse anti-human CD133/1 monoclonal antibody (Cat# 130-092-395, Miltenyi Biotech, Bergisch Gladbach, Germany) as described previously [14]. Sections immunostained for laminin were treated with a 0.125%-trypsin solution (Nichirei Bioscience, Tokyo, Japan) at 37°C for 10 minutes as retrieval, with a primary antibody (a rabbit anti-laminin polyclonal antibody [Cat# L9393, Sigma]), and then with a secondary antibody (a rabbit horseradish peroxidase polymer probe [ChemoMate detection kit, peroxidase/3,3’-diaminobenzidine, rabbit; Dako, Carpinteria, CA]). Immunofluorescent staining was done as described [17].

### TUNEL Assays

KMC14 colonies co-cultured with PA6 cells were incubated with gemcitabine (0, 2.5, 5 and 10 µM in serum-free Stem medium) (Eli Lilly Japan KK, Tokyo, Japan) at 37°C for 48 hours. The cultured cells were fixed in 4% PFA for 5 minutes and harvested, followed by preparation of paraffin-embedded cytology specimens. Deparaffinized and rehydrated 3-µm sections were applied for TUNEL assays using DeadEnd^TM^ Colorimetric TUNEL System (Promega, Madison, WI) according to the manufacture’s protocol.

### Flow Cytometry and Magnetic Cell Separation (MACS)

Pleural effusion was centrifuged and suspended with 1 ml of PBS. KMC14 cells co-cultured with PA6 cells were harvested, dissociated and suspended with 1 ml of PBS. Nonspecific antibody binding was blocked using purified rat anti-mouse CD16/CD32 monoclonal antibodies (Cat# 553142, BD Biosciences) and a human FcR blocking reagent (Cat# 130-059-901, Miltenyi Biotech) on ice for 15 minutes. The cells were incubated with a rat biotin-conjugated anti-mouse PDGFRß monoclonal antibody (Cat# 13-1401-80, eBioscience, San Diego, CA) and a PE-conjugated anti-human CD133 monoclonal antibody (Cat# 130-080-801, Miltenyi Biotech) on ice for 30 minutes and re-suspended in PBS containing 0.5% bovine serum albumin and 1 µl of 1g/ml 7-Amino-Actinomycin D (BD Biosciences) to exclude dead cells. The cells were sorted and analyzed by FACS Aria (*n* = 3; Becton Dickinson Immunocytometry Systems, BD Biosciences)[17].

For MACS, the cells after blocking were separated by MACS Separator (Miltenyi Biotech) using a biotin-conjugated anti-mouse PDGFRß monoclonal antibody and biotin Beads, subsequent human CD133 MicroBeads Kit (Cat# 130-050-801, Miltenyi Biotech) according to the manufacture’s protocol. Purities ranged from 95 to 98 % for each cell population, evaluated by FACS analyses (data not shown).

### Engraftment of KMC14 Cells and Standard Limited Dilution Assays

About 8 week-old male nude mice (BALB/cAJc1-nu/nu) (CLEA, Tokyo, Japan) were used. All nude mice were housed and used under the approved protocols in accordance with the Kobe University guidelines for the care and use of laboratory animals (Permit Number: P120601). All surgery was performed under Avertin, and all efforts were made to minimize suffering. After a small left abdominal flank incision was made, 200 µL of a single KMC14-cell suspension (1 x 10^4^ KMC14 cells in 200 µL of serum-free Stem medium), PA6 cells (1 x 10^5^ cells in 200 µL of serum-free Stem medium), or admixture of KMC14 and PA6 cells (1 x 10^4^ KMC14 cells and 1 x 10^5^ PA6 cells in 200 µL of serum-free Stem medium) were injected in a region of the pancreas tail using a 1-mL disposable syringe attached to a 30-gauge needle (TERUMO, Tokyo, Japan). One layer of the abdominal wound was closed with Auto-clip (Clay Adams, Parsippany, NJ). After 8 weeks, the mice were sacrificed under anesthesia. Tissue samples were fixed in 10% phosphate-buffered formalin for overnight and embedded in paraffin.

KMC14 cells separated from mouse PA6 cells expressing mouse PDGFRß human CD133^+^/mouse PDGFRß^–^ KMC14 cells and human CD133^-^ /mouse PDGFRß^–^ KMC14 cells were prepared by MACS Separator. The separated cells were mixed with 100 µL of Matrigel (Cat# 356234, BD Matrigel™, BD Biosciences) and subcutaneously injected into nude mice (1 x 10^2^ cells, 1 x 10^3^ cells and 1 x 10^4^ cells per injection). Mice were examined for tumor formation by palpation and a subsequent autopsy.

### Transwell Co-Culture Assays

PA6 cells were seeded in the lower well of a transwell culture system (24-well type, PET track-etched membranes with 0.4-µm pores; BD Biosciences) and grown to confluence and then the culture medium was re-placed with serum-free Stem medium. A single KMC14-cell suspension (2 x10^2^ cells) was plated in the upper chamber (a cell culture insert). Four weeks later, colonies were observed under a microscope.

### Reverse Transcription (RT)-Polymerase Chain Reaction (PCR) and Quantitative Real Time (qRT)-PCR Assays

RT-PCR was performed as described [17]. qRT-PCR was performed with a QuantiTect SYBR Green RT-PCR kit (Qiagen) using Applied Biosystem 7500 Real-Time PCR System (Life Technologies, Tokyo, Japan) according to manufactures’ protocols. Quantification of gene expression was determined by the relative standard curve method with *human ß-actin* and *mouse cyclophilin A* as endogenous controls. The primer sequences used were described in Table 2.

**Table 2:** List of primer sequences.

**Human primers**		Sequence (5'->3')		Product length	
CD133 (001145852.1)	Forward	TGCCCAGGAACACGCTTGCC		115	
	Reverse	GGAGCCGAGTACGAGGGCCA			
ß-Actin (NM_001101.3)	Forward	AGCCTCGCCTTTGCCGATCC	104		
	Reverse	TTGCACATGCCGGAGCCGTT			
Notch-1 (NM_017617.3)	Forward	CCGCAGTTGTGCTCCTGAA	109		
	Reverse	ACCTTGGCGGTCTCGTAGCT			
Notch-2 (NM_024408.3)	Forward	GGTGACTCTCTGCCCTTGGACC	98		
	Reverse	GGCAGAAAGGGGCAGGTACGC			
Jagged-1 (NM_000214.2)	Forward	CTGCCTCTCTGATCCCTGTC	76		
	Reverse	TGGGGAACACTCACACTCAA			
Jagged-2 (AF003521.1)	Forward	GCCCGCTACGTCGGCAAGGAATAG	77		
	Reverse	AGACGGCATGGCTCCCACCGAG			
c-Met (NM_000245.2)	Forward	GCTTCATGCAGGTTGTGGTTTC	156		
	Reverse	ATCTTCGTGATCTTCTTCCCAGTG			
CXCR-4 (NM_003467.2)	Forward	CCGCATCTGGAGAACCAGCGG	84		
	Reverse	CCCCTGAGCCCATTTCCTCGG			
CD24 (NM_013230.2)	Forward	TGAAGAACATGTGAGAGGTTTGAC	208		
	Reverse	GAAAACTGAATCTCCATTCCACAA			
CD44 (NM_000610.3)	Forward	TACAGCATCTCTCGGACGGA	108		
	Reverse	GCAGGTCTCAAATCCGATGC			
**Mouse primers**		Sequence (5'->3')			Product length
Laminin gamma-1 (NM_010683.2)	Forward	GCGCGATCTTGGCTCGGACG			114
	Reverse	CGGCGTATCCCGAGGCCGAT			
Laminin alpha-5 (NM_001081171.2)	Forward	CAACAGCAACAAGGCACACCCTGT			104
	Reverse	CGTTGACCTCATTGTACTCCAGGCC			
Collagen type-I alpha-1 (NM_007742.3)	Forward	GGTCCCCAATGGTGAGACGTGG			81
	Reverse	ACGTCATCGCACACAGCCGT			
Collagen type-III alpha-I (NM_009930.2)	Forward	AGAAAGGGGCTGGAAAGTGAGGGA			98
	Reverse	CAGCACCGGGCCCGTCATAAAA			
Collagen-IV alpha-I (J04694.1)	Forward	CCAAAGGTGCTAGGGGCGTGAGG			78
	Reverse	AGCACTCACATCTGGGGTGACAGG			
Fibronectin-I (NM_010233.1)	Forward	TGGACTCCACCTCGAGCCCG			143
	Reverse	GCGGTGTACTCAGACCCAGGC			
Integrin alpha-3 (NM_013565.2)	Forward	AGCTAACACCTCCAAGGGGGCG			83
	Reverse	TTCGGTGGACGGGTCACCGTT			
Cyclophilin A (NM_008907.1)	Forward	TGCACTGCCAAGACTGAATGGCTG			100
	Reverse	TGGACCCAAAACGCTCCATGGC			

### Synthesized Basement Membrane (sBM)

A sBM was prepared with some modification according to the previous reports [20,21]. Briefly, a stiff matrix of type I collagen gel (Koken, Tokyo, Japan) was prepared on six-well culture inserts with PET porous membrane with a 0.8 µm diameter pore size (BD Biosciences). PA6 cells were seeded on the collagen and cultured in αMEM containing 10% fetal calf serum (FCS). A single KMC14-cell suspension was seeded on confluent PA6 cells and cultured with serum-free Stem medium. The secreted extracellular matrix molecules were integrated to form a lamina dense structure beneath PA6 cells. Cells were removed with PBS containing 50 mM NH_4_OH, 0.1% Triton X-100 and protease inhibitor cocktail (Roche). Condition medium (CM) was collected from co-culture dishes in the presence of KMC14 and PA6 cells and filtered using a 0.45-µm filter (Merck Millpore, Billerica, MA). The isolated KMC14 colonies were cultured on sBM in CM. Half of the CM was changed every 3 days with fresh CM for 2 weeks.

### Cell Doubling Time

Human CD133^+^/mouse PDGFRß^–^ KMC14 cells (2 x 10^3^ cells/well) and human CD133^-^/mouse PDGFRß^–^ KMC14 cells (2 x 10^3^ cells/well) prepared by MACS Separator were plated on confluent PA6 cells in 6-well plates (9.6 cm^2^/well) and cultured in serum-free Stem medium. The culture medium was changed every 7 days. PDGFRß^–^ KMC14 cells prepared by MACS Separator were counted at 24 h intervals for 20 days in duplication. The doubling time of the KMC14 cell population was calculated from the logarithmic growth curve by the following formula as described [22]: 

v=logN – logN0/log2(t-t0), with doubling time=1/v 

### Statistical Analysis

Results for continuous variables were expressed as means ± standard error (SE). Statistically significant differences were determined by Student’s *t*-Test. Significance was defined as *, *p* < 0.05, and **, *p* < 0.005.

## Results

### Isolation of Tumor-Initiating KMC14 Cells

**Figure 1:**
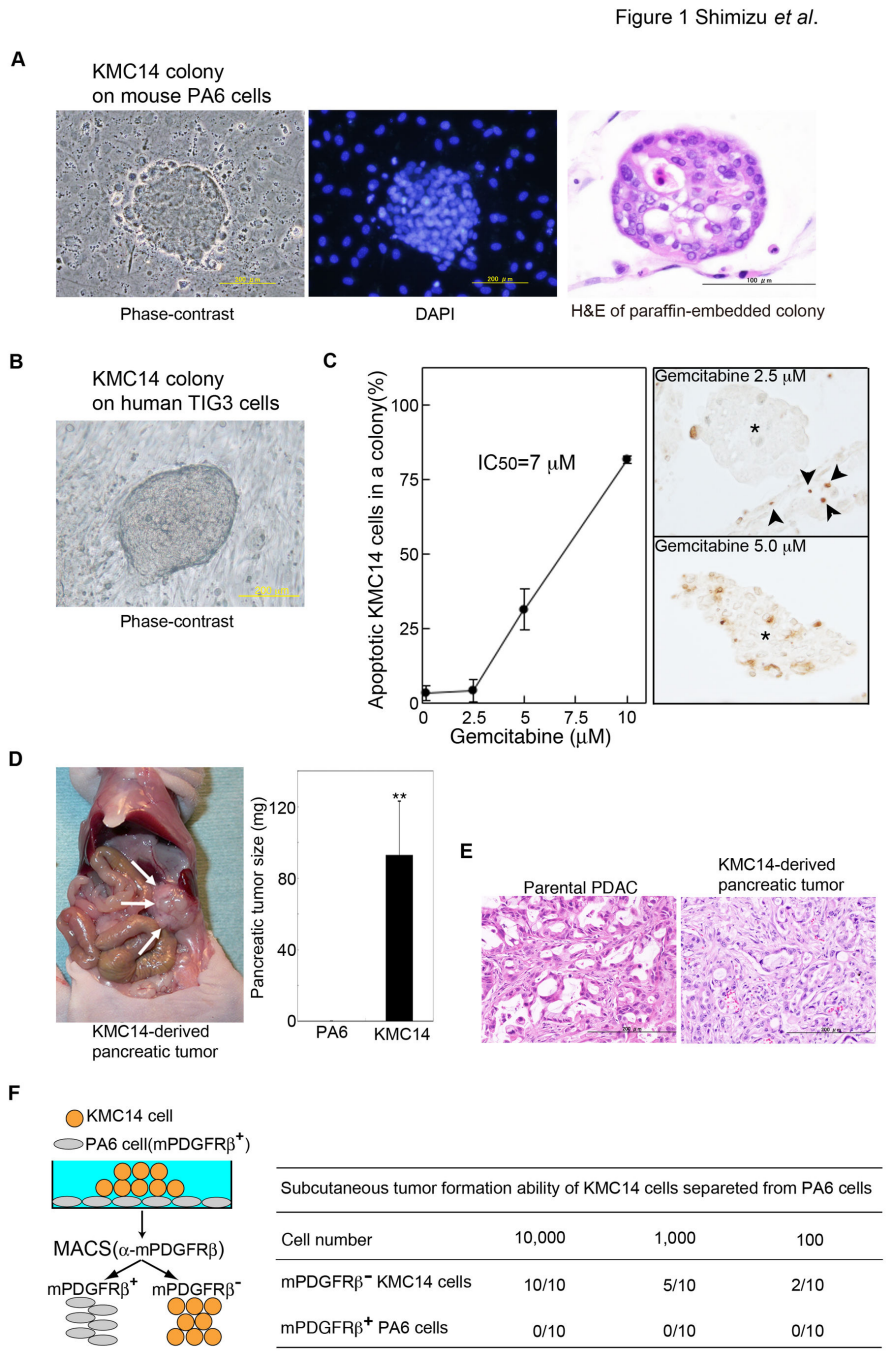
Isolation of pancreatic tumor-initiating KMC14 cells. **A**. A colony-forming property of KMC14 cells on PA6 cells. Scale bar = 200 µm (phase-contrast and DAPI); 100 μm (H&E staining). **B**. A colony-forming property of KMC14 cells on TIG3 cells. Scale bar = 200 µm. **C**. Gemcitabine resistance of KMC14 cells. KMC14 cells co-cultured with PA6 cells were treated with indicated concentrations of gemcitabine, followed by TUNEL assays. The left panel shows the percentage of apoptotic KMC14 cells in a colony. The values are mean of three experiments ± SE completed in triplicate. The right panels are representatives of three independent experiments. Asterisks indicate KMC14 colonies. Arrowheads indicate apoptotic PA6 cells. **D**. Pancreatic tumor formation by KMC14 cells. The left panel shows a xenograft tumor formed at the pancreas tail of a nude mouse. Arrows point to the tumor. The right panel shows the pancreatic tumor weight. Columns, mean of three experiments ± SE completed in triplicate; **, *p* < 0.005. **E**. Histology of KMC14-derived pancreatic tumor. PDAC tissues from the patient 1 (parental PDAC) and tumor tissues in Figure 1D (KMC14-derived pancreatic tumor) were examined by H&E staining. The result is representative of three independent experiments. Scale bar = 200 µm. **F**. Standard limited dilution assays for *in* *vivo* tumorigenicity of KMC14 cells. KMC14 cells separated from PA6 cells by MACS Separator were injected into the subcutaneum of nude mice at 1 x 10^2^ cells, 1 x 10^3^ cells and 1 x 10^4^ cells per injection. After 7 weeks mice were examined for xenograft tumor formation. Data are expressed as number of tumors formed/number of injections.

We obtained pleural effusions from a gemcitabine-resistant PDAC patient (Table 1, [patient 1]) and prepared the cell fraction containing carcinoma cells. When the cell fraction was cultured on a low-attachment plate or a culture dish with serum-free Stem medium or DMEM containing 10% FCS, there were neither tumor-spheres nor adhesive cells observed (data not shown). We then cultured the cell fraction on PA6 cells with serum-free Stem medium and observed dome-like KMC14 colonies (Figure 1A). The percentage of colony-forming cells in the cell fraction was 0.001% ± 0.0008%. The clonogenic activity of KMC14 cells in the first, the second and the third passages was 14% ± 3.1%, 15% ± 2.7%, and 12 ± 3.8%, respectively (studied up to 3 passages). KMC14 cells formed the similar dome-like colonies on human TIG3 cells to those on mouse PA6 cells (Figure 1B). TUNEL assays showed that KMC14 cells were resistant to gemcitabine with approximately 7 µM of IC_50_ (Figure 1C). To evaluate the *in vivo* tumorigenicity of KMC14 cells, KMC14 colonies co-cultured with PA6 cells were hand picked and orthotopically implanted into pancreases of nude mice. KMC14 cells formed pancreatic tumors with a mean tumor weight of 93.0 mg ± 31.0 mg (Figure 1D). On the other hand, PA6 cells alone did not form any tumor. Co-injection of KMC14 and PA6 cells resulted in pancreatic tumor formation to the same extent as KMC14 cells alone (data not shown). Histologically, the pancreatic tumors derived from KMC14 cells resembled the patient’s primary PDAC (parental PDAC) (Figure 1E). PA6 cells expressed mouse PDGFRß (mPDGFRß) (data not shown). We excluded PA6 cells from the KMC14 cell fraction by MACS Separator using anti-mPDGFRß monoclonal antibody and subcutaneously injected the mPDGFRß^–^ KMC14 cells in nude mice for standard limited dilution assays as described [9,23]. We found that 2 of 10 animals, 5 of 10 animals and 10 of 10 animals injected with 1 x 10^2^, 1 x 10^3^ and 1 x 10^4^ mPDGFRß^–^ KMC14 cells formed tumors in the subcutaneous fat of nude mice, respectively (Figure 1F). These results demonstrate that KMC14 cells are indeed pancreatic tumor-initiating cells.

### Remodeling of ECM Formation by KMC14 Cells

**Figure 2:**
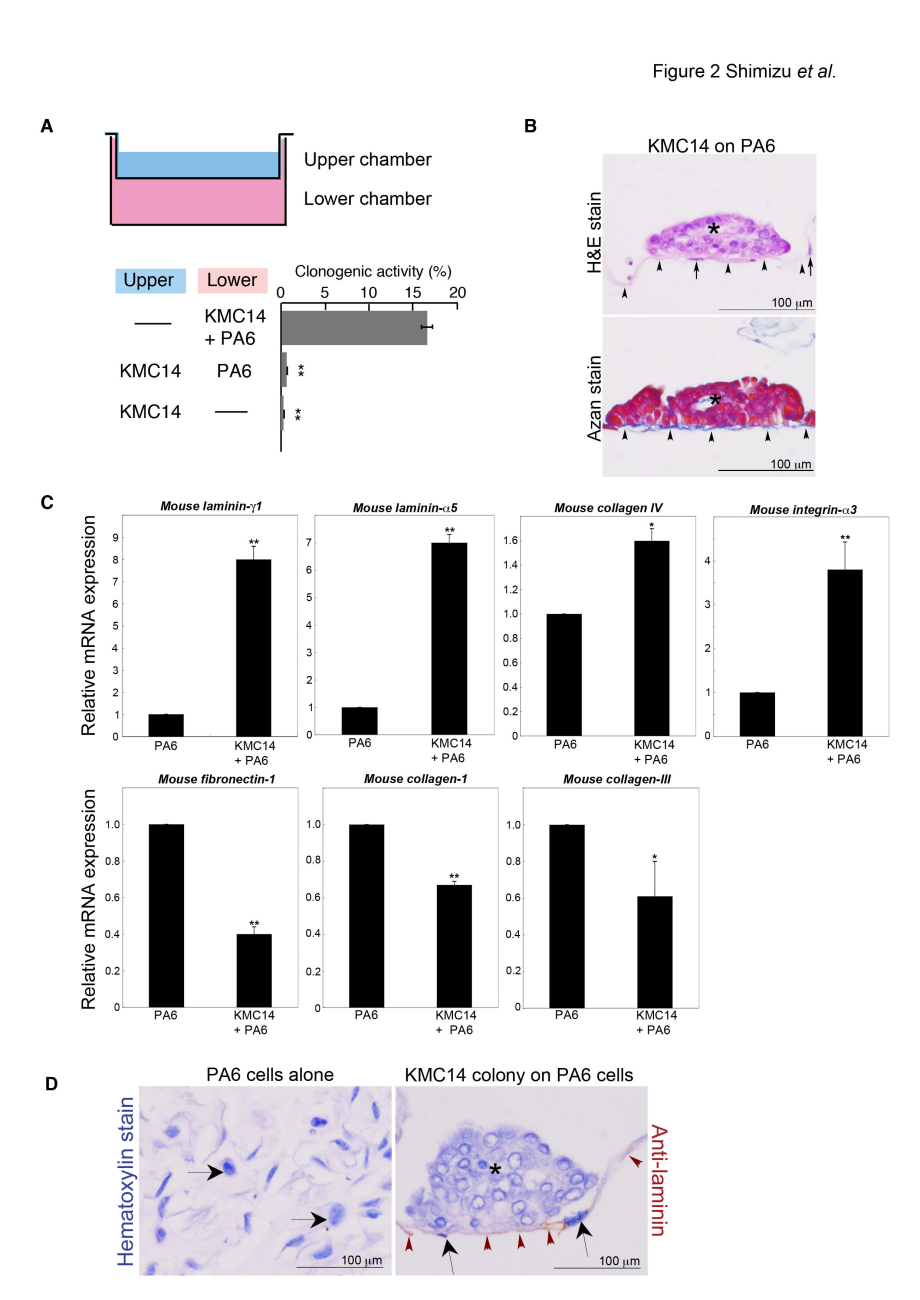
Remodeling of ECM by KMC14 cells. **A**. Transwell co-culture assays. A single KMC14-cell suspension was seeded directly on PA6 cells or in the upper chamber in the presence or absence of PA6 cells in the lower well. Relative colony number/total KMC14 cell number seeded in a well was evaluated. Columns, mean of three experiments ± SE completed in triplicate; **, *p* < 0.005. **B**. ECM formation in the presence of KMC14 cells. The upper panel shows H&E staining. An asterisk indicates a KMC14 colony. Arrows and arrowheads point to PA6 cells and ECM, respectively. The lower panel shows Azan staining. An asterisk indicates a KMC14 colony. Arrowheads point to ECM (blue color). Scale bar = 100 µm. The result is representative of three independent experiments. **C**. qRT-PCR assays of the expression levels of PA6 cell-derived ECM constituents. Confluent PA6 cells were cultured with or without KMC14 cells in serum-free Stem medium, followed by qRT-PCR assays. Results were normalized to *mouse* *cyclophilin* *A* mRNA and to the control condition (PA6 cells alone). Columns, mean of three experiments ± SE completed in triplicate; **, *p* < 0.005; and *, *p* < 0.05. **D**. Enrichment of laminin at the KMC14-induced basal lamina-like structure. Confluent PA6 cells were cultured with or without KMC14 cells in serum-free Stem medium, followed by laminin immunochemical staining. An asterisk indicates a KMC14 colony. Arrows and arrowheads point to PA6 cells and ECM (brown color), respectively. Scale bar = 100 µm. The result is representative of three independent experiments.

Transwell co-culture assays showed that KMC14 cells required direct interactions with PA6 cells for their clonogenic activity (Figure 2A). Confluent PA6 cells were easily dissociated to single cells by trypsin-EDTA treatment, whereas confluent PA6 cells co-cultured with KMC14 cells were detached as “a sheet” from a dish by trypsin-EDTA treatment, which was hardly dissociated to single cells (data not shown). We observed that KMC14 cells formed dome-like colonies on thick and bundled collagen filaments, which looked like basal lamina (Figure 2B). The mRNA expression levels of *mouse laminin-gamma1* and *-*α*5* and *collagen-IV*, major components of basal lamina, and *mouse integrin-*α3 were increased in the presence of KMC14 cells, while those of *mouse collagen type-I* and type-*III* and *fibronectin-I* were reduced in the presence of KMC14 cells (Figure 2C). Laminin was enriched at the basal lamina-like structure attaching to the basal side of dome-like KMC14 colonies (Figure 2D).

**Figure 3:**
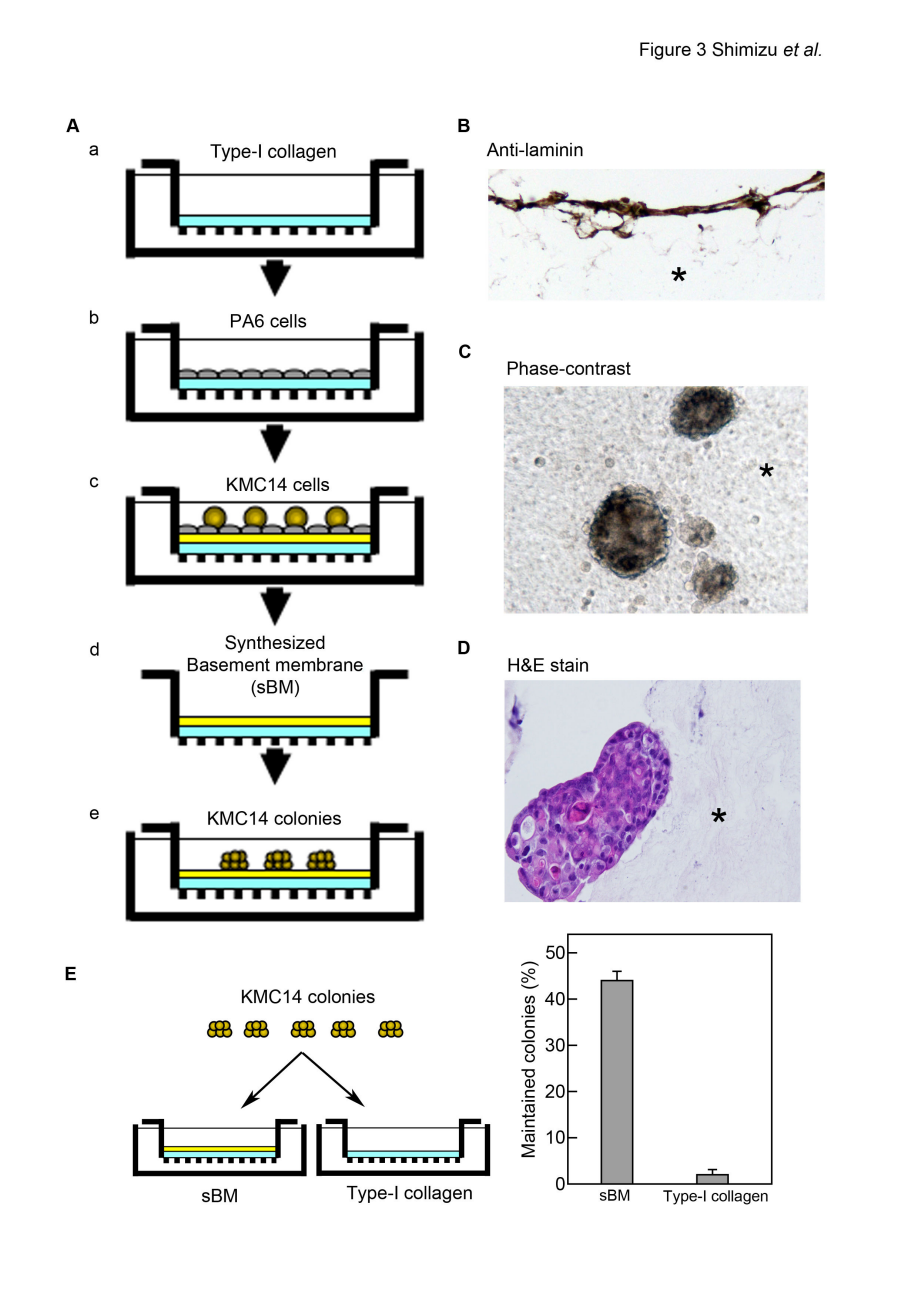
Maintenance of KMC14 colonies by sBM. **A**. A schema of the sBM preparation procedure. Type-I collagen gel (light blue) was on a cell culture insert (a), where PA6 cells were seeded (gray) (b). KMC14 cells (ocher) were seeded on PA6 cell sheets and allowed to form the BM (yellow) (c). KMC14 and PA6 cells were removed (d). Isolated KMC14 colonies were seeded on the sBM and cultured (e). **B**. Structure and component analyses of sBM. sBM was immunostained for laminin. Brown staining indicates a laminin-based ECM. An asterisk indicates a stiff matrix of type-I collagen. Original magnification, 200x. The result is representative of three independent experiments. **C**-**E**. Maintenance of KMC14 colonies by sBM. KMC14 colonies were seeded on sBM, cultured for 2 weeks and observed. (**C**) Horizontal viewing and (**D**) transverse (vertical) sections of KMC14 colonies on sBM; asterisks, sBM. Original magnification, 200x. The results are representative of three independent experiments. (**E**) The number of KMC14 colonies on sBM or stiff matrix of type-I collagen gel was counted. Columns, mean of three experiments ± SE completed in triplicate.

We prepared sBM (Figure 3A) and found that laminin was expressed in the sBM (Figure 3B). KMC14 colonies were maintained on the sBM, but not on type-I collagen (Figures 3, C-E). When a single KMC14-cell suspension was cultured on the sBM, there was no colony observed (data not shown). These results demonstrate that KMC14 cells induce basal lamina-like ECM formation via stromal cells that maintains KMC14 colony formation. 

### CD133 Expression in KMC14 Colonies and KMC14-Derived Pancreatic Tumors

**Figure 4:**
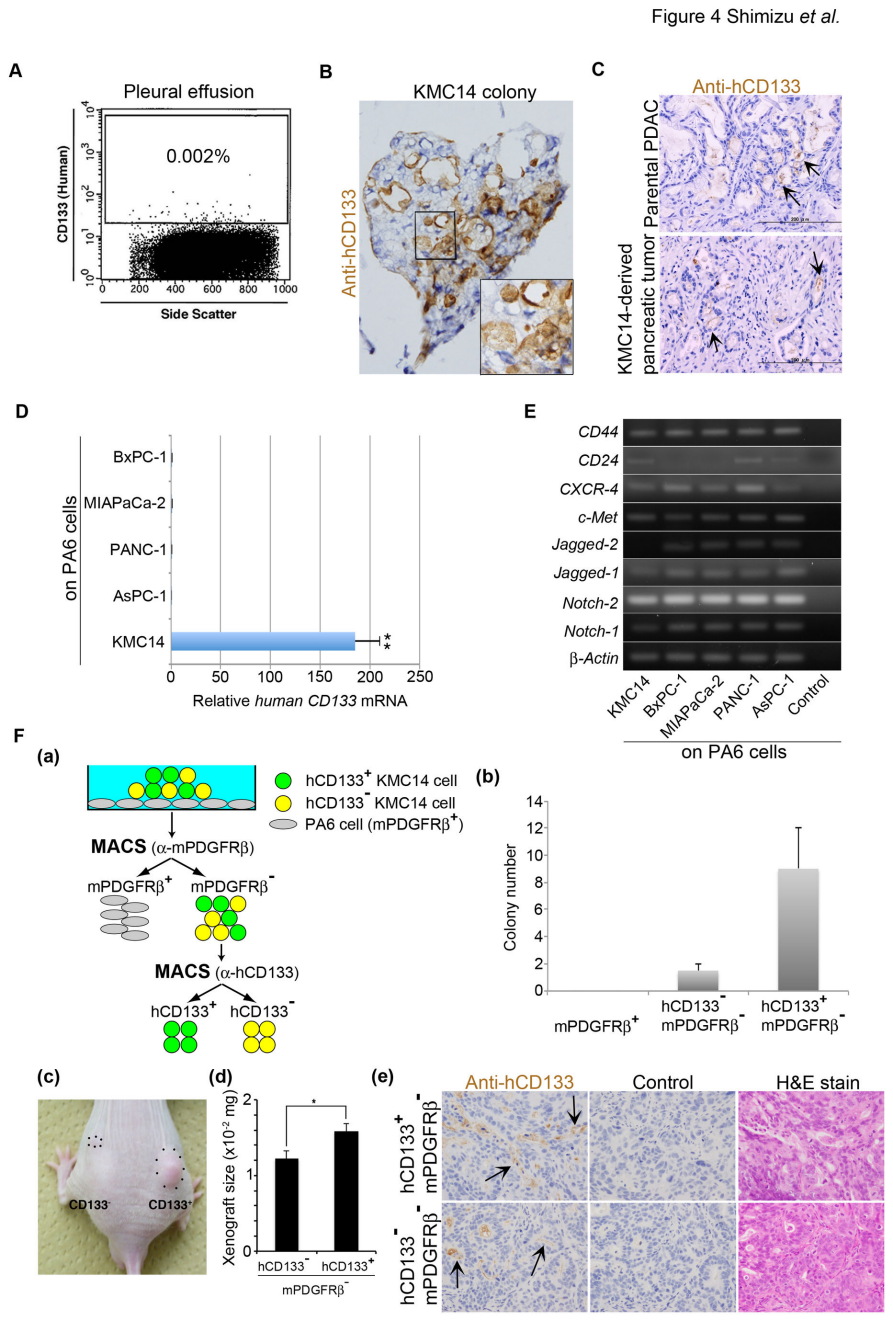
CD133 expression in KMC14 cells. **A**. Flow cytometric analyses for CD133^+^ cells in pleural effusions derived from patient 1. The result is representative of three independent experiments. **B**. CD133 expression in KMC14 cells. Frozen sections of KMC14 colonies were immunostained for human CD133 (hCD133) (brown color). The boxed region (magnification, x100) is shown at higher magnification in the insert (magnification, x400). The result is representative of three independent experiments. **C**. CD133 expression in KMC14-derived pancreatic tumor. PDAC tissues from the patient 1 (parental PDAC) and KMC14-derived pancreatic tumor tissues in Figure 1E were immunostained for hCD133 (brown color). Arrows indicate hCD33^+^ carcinoma cells. Scale bar = 200 µm. The result is representative of three independent experiments. **D**. *CD133* mRNA expression analyses of each cell line by qRT-PCR assays. Confluent PA6 cells were cultured with KMC14, BxPC-1, MIAPaCa-2, PANC-1 or AsPC-1 cells in serum-free Stem medium, followed by qRT-PCR assays. Results were normalized to *human* *ß-actin* mRNA and to the control condition (PANC-1 cells). Columns, mean of three experiments ± SE completed in triplicate, **, *p* < 0.005. **E**. Gene expression analyses of each cell line by RT-PCR. Confluent PA6 cells were cultured with KMC14, BxPC-1, MIAPaCa-2, PANC-1 or AsPC-1 cells in serum-free Stem medium, followed by qRT-PCR assays. Control: PA6 cells alone. *Human* *ß-actin* was used as an endogenous control. The result is representative of three independent experiments. **F**. Effect of hCD133 expression in KMC14 cells on their clonogenic activity and xenograft tumor formation. (a) Scheme of separation of mPDGFRß^+^ PA6 cells and hCD133^+^ and hCD133^-^ mPDGFRß^–^ KMC14 cells from the co-culture system in serum-free Stem medium. (b) Comparison of clonogenic activity between hCD133^+^ and hCD133^-^ mPDGFRß^–^ KMC14 cells. mPDGFRß^+^ PA6 cells, and hCD133^+^ and hCD133^-^ mPDGFRß^–^ KMC14 cells were co-cultured with PA6 cells in serum-free Stem medium for 2 weeks and the number of colonies were counted. Columns, mean of three independent experiments ± SE completed in triplicate. (c) Comparison of xenograft tumor formation between hCD133^+^ and hCD133^-^ mPDGFRß^–^ KMC14 cells. hCD133^+^ and hCD133^-^ mPDGFRß^–^ KMC14 cells were subcutaneously injected into the right and left back of nude mice at 1 x 10^4^ cells per injection. After 7 weeks mice were examined for xenograft tumor formation. The result is representative of five independent experiments. (d) Xenograft tumor size. Xenograft tumors formed subcutaneously in Fig. 4Fc were dissected and weighted. Columns, mean of five experiments ± SE; *, *p* < 0.05. (e) hCD133 expression in xenograft tumors derived from hCD133^+^ and hCD133^-^ mPDGFRß^–^ KMC14 cells. The xenograft tumor tissues in Fig. 4Fc were examined by H&E staining and immunostaining for hCD133 (brown color). The result is representative of five independent experiments. Control: PBS instead of anti-hCD133 monoclonal antibody. Arrows indicate hCD133^+^ carcinoma cells. Original magnifications: 400x.

Flow cytometric analyses showed that an average of 0.002% of cells in the pleural effusion containing KMC14 cells was human CD133^+^ (hCD133^+^) (Figure 4A). There were both hCD133^+^ and hCD133^-^ KMC14 cells in a colony and hCD133 staining was observed at the plasma membrane and cytoplasm of hCD133^+^ KMC14 cells (Figure 4B). We confirmed that KMC14-derived pancreatic tumors as well as the patient’s primary PDAC (parental PDAC) expressed hCD133 protein (Figure 4C) as described previously [14]. qRT-PCR assays showed that expression level of *CD133* mRNA in KMC14 cells was much higher than those in BxPC-1, MIAPaCa-2, PANC-1 and AsPC-1 cells in the co-culture system (Figure 4D). hCD133 staining was not observed in BxPC-1, MIAPaCa-2, AsPC-1 or PANC-1 cells in the co-culture system (data not shown). KMC14 cells expressed mRNA of Notch-1, Notch-2, Jagged-1, c-Met, CXCR-4, CD24 and CD44, except Jagged-2, that were reported as pancreatic cancer markers (Figure 4E) [9,23-26]. These results demonstrate that KMC14 cells express hCD133 protein and its expression level varies in a KMC14 colony.

### Effects of CD133 Expression on KMC14-Derived Colony and Tumor Formation

We first separated KMC14 cells from PA6 cells expressing mPDGFRß and then separated hCD133^+^ KMC14 cells from hCD133^-^ KMC14 cells by MACS Separator using anti-mPDGFRß and anti-hCD133 monoclonal antibodies (Fig. 4Fa). hCD133^+^ and hCD133^-^ KMC14 cells were co-cultured with PA6 cells. Both the cell types formed colonies, but the clonogenic activity of hCD133^+^ KMC14 cells was higher than that of hCD133^-^ KMC14 cells (Fig. 4Fb). mPDGFRß^+^ PA6 cells did not form any colony. The doubling times of KMC14 cells in hCD133^+^ and hCD133^-^ KMC14-derived colonies were 41.9 and 55.6 hours, respectively. Both CD133^+^ and CD133^-^ KMC14 cells appeared in a colony derived from an hCD133^+^ KMC14 cell (data not shown). Standard limited dilution assays resulted in xenograft tumor formation of 5 of 5 and 5 of 5 animals, 1 of 5 and 4 of 5 animals, and 1 of 5 and 1 of 5 animals subcutaneously injected with 1 x 10^4^, 1 x 10^3^ and 1 x 10^2^ hCD133^+^ and hCD133^-^ KMC14 cells into the right and left back, respectively. Although both hCD133^+^ and hCD133^-^ KMC14 cells formed xenograft tumors at the similar rate, initial velocity of hCD133^+^ KMC14-derived tumor formation was higher than that of hCD133^-^ KMC14-derived tumor formation (Figs. 4Fc and 4Fd). Histologically, xenograft tumors derived from hCD133^+^ KMC14 cells resembled those from hCD133^-^ KMC14 cells (Fig. 4Fe). Xenograft tumors derived from hCD133^+^ KMC14 cells expressed hCD133, and surprisingly those derived from hCD133^-^ KMC14 cells also expressed hCD133 (Fig. 4Fe). The hCD133^+^ cell population in hCD133^+^ and hCD133^-^ KMC14-derived carcinoma cells was 40.2% ± 8.3% and 30.7% ± 8.79% (*p*=0.245), respectively. Our findings suggest that CD133 expression affects the clonogenic activity and initial velocity of the xenograft tumor formation.

### Effect of Culture Conditions on the CD133 Expression Level in KMC14 Cells

**Figure 5:**
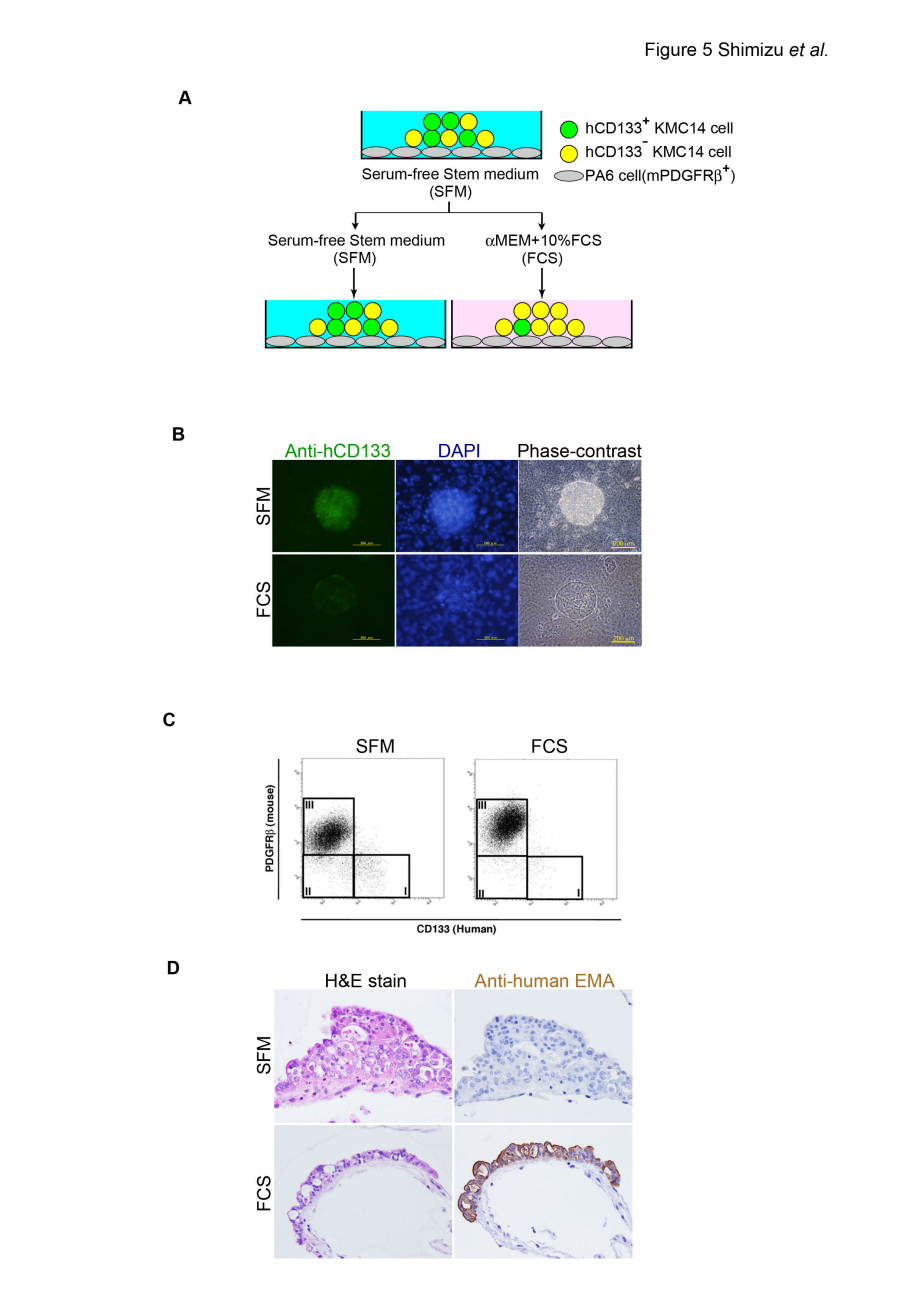
Effect of culture conditions on CD133 expression and morphology of KMC14 colonies. **A**. Scheme of culture medium switch experiments. **B**. CD133 immunofluorescent staining of KMC14 colonies. KMC14 colonies on PA6 cells were incubated in serum-free Stem medium (SFM) or αMEM containing 10% FCS (FCS), followed by hCD133 immunofluorescent staining. The result is representative of three independent experiments. Scale bar = 200 µm. **C**. Flow cytometric analyses for hCD133^+^ KMC14 cells in the co-culture system. KMC14 colonies on PA6 cells were incubated in serum-free Stem medium (SFM) or αMEM containing 10% FCS (FCS), followed by flow cytometric analyses. I: hCD133^+^ mPDGFRß^-^ cells (hCD133^+^ KMC14 cells); II, hCD133^-^ mPDGFRß^-^ cells (hCD133^-^ KMC14 cells); and III, mPDGFRß^+^ cells (PA6 cells). The result is representative of five independent experiments. **D**. Morphology change of KMC14 colonies. KMC14 colonies co-cultured with PA6 cells were incubated in serum-free Stem medium (SFM) or αMEM containing 10% FCS (FCS), followed by H&E staining and EMA immunostaining (brown color) (transverse sections). Original magnification, 200x. The result is representative of three independent experiments.

When KMC14 colonies were cultured in αMEM containing 10% FCS, the staining intensity of hCD133 in KMC14 cells was reduced (Figures 5A and 5B). Flow cytometric analyses showed that the percentage of hCD133^+^ mPDGFRß^–^ KMC14 cells in total KMC14 cells (mPDGFRß^-^ cells) decreased from 43.1% to 20.9% when culture medium was changed from serum-free Stem medium to αMEM containing 10% FCS (Figure 5C). Moreover, in αMEM containing 10% FCS KMC14 colonies changed their morphology from a dome-like to cobblestone-like phenotype (Figures 5B and 5D). The cobblestone-like colonies consisted of duct-like structures expressing epithelial membrane antigen (EMA) (Figure 5D). These results demonstrate that the culture conditions affect the expression level of CD133 and morphology of KMC14 colonies. 

### A KMC14 Cell-Like Subpopulation in Gemcitabine-Resistant PDAC Patients

We applied the KMC14-cell isolation method for other 6 PDAC patients who resisted gemcitabine, and established 6 CD133^+^ tumor-initiating cells that required stromal cells for their *in vitro* clonogenic activity (Tables 1 and 3). These cell lines remodeled ECM and expressed CD133, suggesting that there is a KMC14 cell-like subpopulation in gemcitabine-resistant PDAC.

**Table 3:** Summary of characteristics of KMC cells.

	*In vitro* clonogenic activity	*In vivo* tumorigenicity	Requirement of stromal cells	ECM remodeling	CD133 expression
KMC14	+	+	+	+	+
KMC16	+	+	+	+	+
KMC17	+	+	+	+	+
KMC18	+	+	+	+	+
KMC26	+	+	+	+	+
KMC07	+	+	+	+	+
KMC09	+	+	+	+	+

*In vitro* clonogenic activity: the capability of dome-like colony formation in co-culture system with PA6 cells in serum-free Stem medium. *In vivo* tumorigenicity: the capability of *in vivo* tumor formation at pancreases of nude mice. Requirement of stromal cells: requirement of PA6 cells for the clonogenic activity. ECM remodeling: the capability to remodel ECM formation. CD133 expression: expression of mRNA and protein of CD133.

## Discussion

In this study, we established a KMC14 cell line, a novel pancreatic tumor-initiating cell subpopulation, from disseminated cancer cells of gemcitabine-resistant PDAC patients. To our knowledge, this is the first report demonstrating the establishment of primary culture of gemcitabine-resistant PDAC using stromal cell lines. Moreover, we established the similar cell lines from other 6 gemcitabine-resistant PDAC patients to KMC14 cells. Further characterization of KMC14 cells and other KMC14-like cells will help to develop novel therapies that enhance effects of gemcitabine or novel anti-cancer drugs.

 KMC14 cells required direct interactions with stromal cells for their clonogenic activity. KMC14 cells were capable to remodel ECM formation. The remodeled basal lamina-like ECM was enough to maintain KMC14 colonies, but not to induce formation of KMC14 colonies, suggesting that some unidentified factors regulated by the direct interactions between KMC14 and stromal cells are required for induction of KMC14 colony formation. The precise function of laminin in pancreatic tumor-initiating cell niche remains unclear. Therefore, to clarify relationship between KMC14 cells and laminin would develop a new therapeutic strategy against microenvironment of gemcitabine-resistant PDAC. KMC14 cells required either mouse or human stromal cells for their *in vitro* clonogenic activity, but not for *in vivo* tumor formation. The reason for the difference might be existence of *in vivo* stromal cells. KMC14 cells and most of KMC14-like cells were established from pleural effusion by carcinomatous pleurisy and ascites by peritonitis carcinomatosa. Our results suggest that KMC14 cells and KMC14-like cells floating in effusions directly interact with normal stromal cells at pleura or peritoneum and form and maintain their colonies through ECM remodeling.

CD133^+^ pancreatic cancer cells were observed in human PDAC [7,13-15]. Isolation of KMC14 cells validates the existence of CD133^+^ pancreatic cancer cells at least in gemcitabine-resistant PDAC. Although CD133 expression was reported to correlate with cancer stem cell-like characteristics of pancreatic cancer cells [7,13], the functional roles of CD133 in cancer cells remains unclear [27-36]. We found that a KMC14 colony consisted of both CD133^+^ and CD133^-^ KMC14 cells in the co-culture system with serum-free Stem medium. Moreover, CD133^-^ KMC14 cells appeared in the colony derived from a CD133^+^ KMC14 cell. The results indicate that KMC14 cells become heterogeneous during colony formation. We observed that both CD133^+^ and CD133^-^ KMC14 cells formed colonies *in vitro* and xenograft tumors *in vivo*, but the clonogenic activity and initial velocity of xenograft tumor formation of CD133^+^ KMC14 cells were higher than those of CD133^-^ KMC cells. These results suggest that CD133 expression in KMC14 cells affects initial velocity of KMC14-derived colony and tumor formation. When KMC14 cells were cultured in αMEM containing 10% FCS, the expression level of CD133 decreased and the colonies changed to a cobblestone-like phenotype that consisted of duct-like structures. EMA was expressed mainly on duct-like structures in PDAC [37], and pathologically differentiated adenocarcinoma consists of duct-like structures. Therefore, KMC14 cells might change to a differentiated phenotype under the culture condition in which the CD133 expression level decreases in αMEM containing 10% FCS. Taken together, these results suggest that CD133 expression in KMC14 cells is dynamically regulated by their microenvironment conditions and that CD133 expression in KMC14 cells affects not only initial velocity of KMC14-derived colony and tumor formation but also differentiation of KMC14 cells. This is one of the reasons why CD133^+^ carcinoma cells were observed in CD133^-^ KMC14-derived xenograft tumors. In this study, we could not detect CD133 expression in PANC-1 cells co-cultured with PA6 cells in serum-free Stem medium. The recent report demonstrated that PANC-1 cells expressed CD133 protein [38]. The difference is probably because we used the co-culture system with PA6 cells in serum-free Stem medium.

Several *in vitro* and xenograft experiments demonstrated that gemcitabine led to a relative increase in the number of CD133^+^ pancreatic cancer stem cells [7,8,10]. Because KMC14 cells had self-renewal capacity, differentiation ability and *in vivo* tumorigenicity, and expressed pancreatic cancer stem cell markers, it seems possible that KMC14 cells might be a subpopulation of CD133^+^ pancreatic cancer stem cells enriched by gemcitabine. Since *in vivo* and *in vitro* single cell analyses were not performed in this study, more precise experiments are required to determine whether cancer stem cells become enriched in KMC14 cells under established culture condition, and if so, which type of KMC14 cells behaves as pancreatic cancer stem cells. 

KMC14 cells formed dome-like colonies on TIG3 cells as well as on PA6 cells and remodeled ECM formation, indicating that the clonogenic activity of KMC14 cells is not different between mouse and human stromal cells. The reason why we did not use cancer-associated stromal cells was to examine how PDAC cells interact with normal stromal cells at the remote metastatic sites. Based on this study, we plan to investigate interaction of cancer-associated stromal cells with KMC14 cells.
